# Postoperative cognitive dysfunction in older surgical patients associated with increased healthcare utilization: a prospective study from an upper-middle-income country

**DOI:** 10.1186/s12877-022-02873-3

**Published:** 2022-03-16

**Authors:** Patumporn Suraarunsumrit, Chadawan Pathonsmith, Varalak Srinonprasert, Nipaporn Sangarunakul, Chalita Jiraphorncharas, Arunotai Siriussawakul

**Affiliations:** 1 Division of Geriatrics, Department of Medicine, Siriraj Hospital, Mahidol University, Bangkok, 10700 Thailand; 2 Department of Medicine, Neurological Institute of Thailand, Bangkok, Thailand; 3 Integrated Perioperative Geriatric Excellent Research Center, Faculty of Medicine, Siriraj Hospital, Mahidol University, Bangkok, 10700 Thailand; 4 Department of Anesthesiology, Faculty of Medicine Siriraj Hospital, Mahidol University, Bangkok, 10700 Thailand

**Keywords:** Functional impairment, Hospital costs, Intensive care units, Mortality, Older patients, Postoperative cognitive dysfunction, Readmission, Surgical patients

## Abstract

**Background:**

Perioperative neurocognitive disorder includes postoperative cognitive dysfunction (POCD) and postoperative delirium (POD). Concerning inconclusive consequences of POCD compared with POD, we explored the association between either POCD or POD and functional decline as well as healthcare utilization.

**Methods:**

Patients aged at least 60 years who underwent a major operation were enrolled. POCD was defined as a decrease in the Montreal Cognitive Assessment (MoCA) score (≥ 2) 1 week after surgery. Postoperative delirium (POD) was defined according to the criteria of the fifth edition of the Diagnostic and Statistical Manual of Mental Disorders (DSM-5). The primary outcome was instrumental activities of daily living (IADLs) 3 months after discharge. Secondary outcomes were the length of stay (LOS), hospital cost, and factors that affected functional decline 3 months after surgery. The multivariate model, including potential confounding factors, namely age, gender, surgery type, and postoperative complications, was used to analyze possible factors that influenced a reduction in function, and the results were expressed by using adjusted relative risk (RR) and 95%CI.

**Results:**

Two hundred eighty-nine patients with a mean age of 72 years were enrolled. The incidence of POCD at 1 week was 28.5%. At their 3-month follow-ups, the patients with POCD were not associated with IADL decline. Nevertheless, patients with POCD were more likely to need a prolonged LOS (11 days [1, 46] vs. 8 days [2, 42]; *P* = 0.01), and incur higher hospital costs (8973.43 USD [3481.69, 11 763.74] vs. 5913.62 USD [332.43, 19 567.33]; *P* < 0.001). Additionally, the patients experiencing POD demonstrated increased risks of reducing their IADLs (adjusted RR 2.33; 95% CI, 1.15–4.71; *P* = 0.02).

**Conclusions:**

POCD at 1 week leaded to increase healthcare utilization in a middle-income country. POD during hospitalization was associated with a decline in function after surgery and increased health care utilization.

**Trial registration:**

Thai Clinical Trials Registry TCTR20190115001.

**Supplementary Information:**

The online version contains supplementary material available at 10.1186/s12877-022-02873-3.

## Background

The number of older patients who undergo surgery has steadily increased due to the progressive aging of the population. Older people are vulnerable to perioperative complications. One of them is perioperative neurocognitive disorder [1], which includes postoperative cognitive dysfunction (POCD), a prolonged state of cognitive impairment that mainly affects higher-level cognitive skills and memory, and postoperative delirium (POD), an acute state of confusion and inattention. POCD was commonly found at approximately 17–43% [2–4], while the POD occurrence accounted for 20–55% [5, 6]. Unlike POD, POCD in terms of diagnosis and its consequences is not known widely [7]. POCD was first described in 1955 [8]. The pathophysiology of the condition has been proposed [9–12]. Neuroinflammation due to peripheral surgical trauma and volatile anesthetics is one of the most reported mechanisms. Furthermore, low intraoperative cerebral oxygenation and cerebral microemboli are other potential mechanistic etiologies. Cognitive deficits from POCD among older patients have been reported to vary, ranging from a few weeks to several years [11, 13, 14].

The diagnosis of POCD is contentious. It has been broadly defined as a drop in cognitive performance in a set of neuropsychological tests administered before and after surgery [15]. However, a variety of tests with different cutoff points have been applied. Considering the definitions of POCD utilized in the research arena, POCD may be underrecognized in some study cohorts and real-life practice because the set of cognitive batteries is not routinely examined either preoperatively or postoperatively. Furthermore, prior to the establishment of nomenclature for cognitive disorders in 2018 [16], subjective cognitive complaints and evidence of functional ability were not included in most POCD studies. In essence, although POCD was detected nearly 70 years ago, the consequences of the condition have yet to be discovered.

Normal cognitive function is necessary to maintain the usual activities of daily living of people. When POCD occurs, it might cause a decline in numerous cognitive domains, mainly the executive function and working memory, for several months. [17]. For this reason, POCD was discovered to be associated with a subsequent functional decline and increased morbidity and mortality after surgery, as shown by some studies [4, 18]. However, there is conflicting evidence for the association of POCD with postoperative function [19, 20]. As to mortality, the presence of POCD was associated with increased mortality in both noncardiac and cardiac surgery [21, 22]. Furthermore, a recent study revealed that the development of POCD was related to an increase in healthcare costs during the 1-year postoperative period [23]. Possible explanations could be differences in several factors: the time points for defining POCD, the diagnostic criteria applied, and the measurements used for functional outcomes. It should be noted that several studies could have reported cognitive disorders after surgery, such as the incidence of POCD, but did not do so. The reports commonly mixed the effects of POCD and POD as the analysis also included POCD with POD [24], and some studies might not have the details of delirium assessment when POCD was documented [22, 25].

In the present study, our objective was to investigate the effects of the association between POCD and functional decline after surgery. We hypothesized that POCD might produce unfavorable outcomes similar to those of POD but to a lesser extent. Therefore, the study explored the relationship between POCD and a reduction in function 3 months after the operation as well as other aspects (length of stay in an intensive care unit (ICU) and hospital, mortality rates, and cost of healthcare). Furthermore, POD and POCD could occur during the same period, so the association between POD and interested outcomes was also investigated. Additionally, factors that affected functional decline 3 months after surgery were also evaluated.

## Materials and methods

### Study design and participants

This prospective cohort study was conducted at a large university hospital and approved by the Siriraj Institutional Review Board, Faculty of Medicine, Siriraj Hospital, Mahidol University, Bangkok, Thailand (Si 515/2017). We recruited Thai-speaking patients over 60 years of age admitted and scheduled for major elective surgery with general anesthesia. "Major surgery" was defined as a procedure involving organ ischemia, high intraoperative blood loss, high noradrenalin requirements, a long operating time, and perioperative blood transfusion. The postoperative factors considered in this study were the systemic inflammatory response and the need for intensive or intermediate care [26]. Patients were excluded if they were unable to undergo cognitive evaluations (for example, due to an inability to communicate in Thai); had severe visual or auditory dysfunction; had significant psychotic disorders affecting cooperation; had preoperative delirium; or were bedridden. We also excluded patients who could not be followed up during the postoperative period. The protocol for this study followed the guidelines of the Declaration of Helsinki and its later amendments. Written informed consent was obtained from all study participants.

### Data collection

Preoperatively, the baseline cognitive statuses of the patients were assessed using the Montreal Cognitive Assessment (MoCA)–Thai version [27] and the modified IQCODE (Informant Questionnaire on Cognitive Decline in the Elderly) [28]. Furthermore, the functional statuses of the patients were determined using the Barthel Index for activities of daily living (Barthel ADL Index) [29] and the Lawton–Brody Instrumental Activities of Daily Living (IADL) Scale [30]. Frailty status was evaluated using the FRAIL Scale (Fatigue, Resistance, Ambulation, Illnesses, and Loss of Weight) [31]. Depressive symptoms were evaluated with the PHQ-9 (the 9-item Patient Health Questionnaire) [32], while the quality of life was assessed with the EQ-5D-5L (the EuroQol 5-level, 5-dimensional questionnaire) [33]. Essential baseline information was collected from the enrolled patients: demographic data, comorbidities, body mass index, and the number of medications used. The baseline characteristics of the patients were obtained from medical records and interviews with the patients and their caregivers. Information relating to comorbid diseases and the types and sites of surgery was drawn from anesthetic records. In addition, a detailed analysis was made of intraoperative and postoperative data (type of surgery, anesthetic technique, operative time, and intraoperative and postoperative complications).

A psychologist visited enrolled participants with cardiac surgery on postoperative days 1 through 5 and those with noncardiac surgery on days 1 to 3. If the patients were medically stable (for example, no acute stroke or hypotension was present), their POD status was evaluated daily using the confusion assessment method for the intensive care unit (CAM-ICU) [34]. CAM-ICU was selected for use for several reasons: firstly, as the tool has formal instructions, healthcare personnel could evaluate postoperative patients similarly. Secondly, some patients were still intubated with respirators on the third postoperative day, so CAM-ICU was more appropriate for screening for POD than CAM. Lastly, healthcare personnel was formally trained in using the CAM-ICU in our setting.

### Cognitive assessments

The cognitive test used in the current work was the MoCA–Thai version. It examines cognitive abilities in 8 domains (visuospatial/executive, naming, memory, attention, language, abstraction, delayed recall, and orientation). Assessments were performed 1 or 2 days before surgery and at 1 week postoperatively (could be any day from D5 to D9). The total possible score was 30, with higher scores representing better performance [35]. The definition of POCD used in this investigation was based on the recommendation of an earlier study [16]. That work considered POCD as present when there was a postoperative decrease of 1 SD or more from the preoperative MoCA score. A previous validation study in the general Thai population reported that the SD of the MoCA was 2.14 [35]. Therefore, we defined POCD as a decrease of 2 or more points in the postoperative MoCA score.

### Assessments to define the perioperative neurocognitive disorder

Figure S1 shows the timeline for assessing participants and defining POCD and POD. Preoperatively, MoCA and CAM-ICU were both administered. On postoperative days 1 through 5, CAM-ICU assessments were carried out daily by a psychologist. When CAM-ICU was positive, a geriatric fellow was notified to conduct a comprehensive clinical evaluation. If the participant exhibits clinical features of delirium according to the criteria of the fifth edition of the Diagnostic and Statistical Manual of Mental Disorders (DSM-5) [36], the diagnosis of POD is made. The MoCA test was administered one week postoperatively (could be any day from D5 to D9 depending upon the discharge planning) to determine their cognitive status. When a participant demonstrated a decline in the MoCA of at least two scores, a geriatric fellow was notified to conduct a clinical assessment. If the participant exhibits clinical features of delirium according to DSM-5 criteria, the diagnosis of POD is also made concomitant with POCD. For the participant with a decline in the MoCA score without POD, POCD was then defined. In case of a drop in MoCA occurred concurrently with POD assessed on a different day, the patient was also defined as having POCD with POD. After the patient was discharged, the medical record was thoroughly reviewed by the geriatrician team to identify any evidence of cognitive and behavioral changes. Whenever evidence in the medical record was sufficient to make a diagnosis of delirium, the POD was also defined.

### Outcome assessments

Three months after their surgeries, the patients visited the postoperative cognitive disorder clinic specifically for a comprehensive evaluation for our project. Their health statuses were determined by a multidisciplinary team (mainly a geriatrician and a psychologist, but other disciplines were involved when appropriate). The geriatrician performed a physical examination and a relevant history of cognition and function. Formal cognitive tests were carried out using the MoCA. Functional evaluations were performed for both basic activities of daily living (BADLs) and instrumental activities of daily living (IADLs). The quality of life related to health was also assessed using the EQ-5D-5L. Additional investigations were carried out according to clinical indications. Additionally, the length of hospital stay, the number of days in an ICU, the number of deceased patients during hospitalization, and hospital costs were also collected.

### Functional assessments

Functional status was assessed using Thai versions of the Barthel ADL Index and the Lawton–Brody IADL Scale. The Barthel ADL Index measures BADLs related to 10 items: feeding, grooming, transferring (bed to chair and back), use of the toilet, mobility, dressing, use of stairs, bathing, bowel control, and bladder control [29]. The Lawton–Brody IADL Scale evaluates 8 IADLs: the ability to use a telephone, shopping, cooking, household chores, laundry, travel, medication management, and personal finance management [30]. The scoring method for the Barthel ADL Index considers whether the person being evaluated receives help while doing each task. The items are also weighted. The resulting BADL range is 0 to 100. The Lawton–Brody IADL Scale scores each item as 0 (the presence of disability) or 1 (no disability), giving an IADL range of 0 to 8. With both instruments, lower scores indicate higher levels of dependency. In this study, IADLs were chosen to assess functional decline because IADLs represent more complex physical and cognitive tasks than BADLs as they are more vulnerable to early pathological events in the brain [37]. A functional decline was defined as a drop of 2 or more points from the preoperative IADL score to that achieved 3 months after surgery.

### Factors associated with functional decline

We evaluated preoperative and postoperative variables that could contribute to functional decline after surgery, namely, an age of 75 years or higher, Charlson comorbidity index, preoperative frailty score, moderate functional dependence at baseline, preexisting cognitive impairment, and baseline depression [38]. In addition, as postoperative complications could be related to reduced function after surgery, those medical complications were collected from medical records (using the 10th revision of the International Statistical Classification of Diseases and Related Health Problems [ICD-10]). For the preoperative variables, moderate functional dependence was defined as Barthel ADL Index scores 0 to 70 [39]. The Thai version of the FRAIL scale consists of 5 items: fatigue (having less energy than in the past), resistance (inability to climb stairs), ambulation (inability to walk 1 block), number of illnesses (more than 5 concurrent illnesses), and loss of weight (> 5% in 1 year) [31]. Since 1 point is assigned to each component, FRAIL scale scores of 3, 4, or 5 are defined as frailty. Furthermore, the PHQ-9 was administered to establish the severity of depressive symptoms [32]. The modified IQCODE for dementia screening was also used to identify significant cognitive changes, with dementia being defined as scores of 3.42 or higher [40].

### Statistical analysis

The sample size calculation was performed using nQuery Advisor 6.0.1 software to investigate the association between POCD and a decrease in IADLs occurring 3 months after surgery in patients undergoing major surgery. Other research found that patients with POCD had an IADL decline of 57.1%, while those without POCD had an IADL decline of 27.3% [41]. With 90% power and a 5% type I error (two-sided), a sample size of 56 subjects with the functional decline was required. In previous studies, the incidence of POCD in noncardiac surgery ranged from 14.1% to 41.1% [4, 41]. Therefore, the incidence of POCD was estimated at approximately 28% for the sample size calculation in this study. A total of 200 participants needed to be enrolled.

Descriptive statistics were used to examine clinical characteristics, perioperative variables, and outcomes of functional decline. Demographic data were reported as numbers and percentages for categorical independent variables (sex, education, American Society of Anesthesiologists [ASA] classification, comorbidity, Lawton–Brody IADL Scale, readmissions, and mortality). Continuous independent variables were represented by the mean ± SD or median (minimum, maximum) for normal and non-normal distributions (age, Charlson comorbidity index, modified IQCODE, MoCA, Barthel ADL Index, hospital LOS, and quality of life).

Regarding the possible factors associated with postoperative functional decline, we evaluated preoperative and postoperative variables that could contribute to functional decline after surgery, namely, an age of 75 years or higher, Charlson comorbidity index, preoperative frailty score, moderate functional dependence at baseline, preexisting cognitive impairment, and baseline depression. In addition, postoperative complications could be related to reduced function after surgery. For univariate analysis, the Chi-square test or Fisher's exact test was used to examine all categorical risk factors. The Mann–Whitney U test or Independent t-test was used to investigate all continuous data. The strength of the association between POCD and POD with functional decline after surgery was assessed using relative risk (RR) and 95% CI. Subsequently, factors with *P* < 0.20 from univariate analysis or clinical meaningful were entered into a multiple logistic regression model of postoperative functional decline, using Poisson models with a log link function and robust standard errors expressed. The strength of the association was presented as adjusted relative risk and 95%CI. *P*-value < 0.05 was considered to indicate statistically significant differences. PASW Statistics for Windows, version 18.0, and Statistic Analysis System (SAS) Studio were employed for the statistical analyses.

## Results

This study enrolled 289 patients who underwent major elective surgery with general anesthesia between December 2017 and February 2020. All were over 60 years of age (mean, 72.32 ± 6.70 years), and the majority (80.3%) had less than 6 years of education. Most participants (76.4%) were ASA class III or IV, 57.8% underwent cardiac surgery, and 50.9% had a Charlson comorbidity index greater than 5. Although most subjects did not have BADL impairment, 27.7% had some impairment in the IADL score (< 8 for women and < 5 for men). As to cognitive status, 14.5% had significant cognitive impairment, according to the modified IQCODE. Based on the comprehensive clinical assessments carried out in the study, it was possible to classify cognitive status after the operations as either POD or POCD with and without POD (Fig. S1). The prevalence of POCD at 1 week was 28.5%. The prevalence of POD was 14.5%, while the prevalence of POCD without POD was 14.9%.

Table 1 presents the baseline characteristics of the patients with and without POCD. Patients with POCD tended to have older age, lower education, higher ASA class, baseline depression, and preoperative frailty compared with those without POCD. However, the differences were nonsignificant. Interestingly, a higher cognitive status was found in the POCD group than in the non-POCD group (mean MoCA scores, 20.86 vs. 18.48; *P* = 0.01). However, there were no significant differences in the other variables of the 2 groups, including the Charlson comorbidity index and preoperative IADL impairment.

**Table 1:** Comparison of baseline characteristics of non-POCD and POCD groups (including only participants who had MoCA administered 1 week after surgery to establish their POCD status)

Variable	No POCD (*n* = 148)	POCD at 1 week (*n* = 59)	*P*-value
Age	72.64 ± 6.48	70.88 ± 7.15	0.09
Male	83 (56.1%)	36 (61.0%)	0.54
Education levels			
6 years or less	114 (77.0%)	49 (83.1%)	0.45
More than 6 years	34 (23.0%)	10 (16.9%)	
BMI	24.33 ± 4.18	24.22 ± 4.32	0.86
ASA class			
Class II	41 (27.7%)	6 (10.2%)	0.01*
Class III	101 (68.2%)	48 (81.4%)	0.06
Class IV	6 (4.1%)	5 (8.5%)	0.30
Charlson comorbidity index	5.78 ± 1.78	5.68 ± 1.92	0.73
Charlson comorbidity index > 5	73 (49.3%)	27 (45.8%)	0.76
Cardiovascular disease	125 (84.5%)	56 (94.9%)	0.06
Endocrine disease	113 (76.4%)	46 (78.0%)	0.86
Respiratory disease	14 (9.5%)	5 (8.5%)	1.00
Nervous system disease	39 (26.4%)	11 (18.6%)	0.28
CKD: 3a, 3b, 4, 5	53 (35.8%)	28 (47.5%)	0.16
Modified IQCODE ≥ 3.42	23 (16.2%)	7 (12.1%)	0.52
MoCA	18.48 ± 5.05	20.86 ± 5.40	0.01*
PHQ-9 ≥ 7	7 (4.7%)	7 (11.9%)	0.12
Barthel ADL Index; score 0–70, moderately disabled	6 (4.1%)	2 (3.4%)	1.00
Barthel ADL Index; score 100	94.52 ± 9.98	94.32 ± 12.77	0.91
Barthel ADL Index; score < 100	60 (41.1%)	24 (40.7%)	1.00
IADL score	6.67 ± 1.70	6.37 ± 2.11	0.33
IADL score < 8 for women or score < 5 for men	37 (25.0%)	15 (25.4%)	1.00
Frailty	15 (10.1%)	12 (20.3%)	0.07
Quality of life at admission	0.85 ± 0.17	0.88 ± 0.17	0.37

Data are presented by n (%) or mean ± SD

*Abbreviations*: *ADL* activities of daily living, *ASA* American Association of Anesthesiologists, *BMI* body mass index, *CKD* chronic kidney diseases, *FRAIL* Fatigue, Resistance, Ambulation, Illnesses, and Loss of weight, *IADL* Lawton–Brody instrumental activities of daily living, *PHQ-9* 9-item Patient Health Questionnaire, *MoCA* Montreal cognitive assessment, *POCD* postoperative cognitive dysfunction

Table 2 compares the demographic data of the non-POD and POD groups. Most patients with POD were more likely to have ASA class III (90.5% vs. 66.8%; *P* = 0.002); a higher mean Charlson comorbidity index (6.55 vs. 5.83; *P* = 0.03); preoperative cognitive impairment (28.2% vs. 12.2%; *P* = 0.01); a lower mean MoCA score (16.76 vs. 19.47; *P* = 0.01); and previous delirium (4.8% vs. 0.0%; *P* = 0.02) than those without POD. Although statistically nonsignificant, the POD group tended to have preoperative IADL impairment and baseline frailty rather than non-POD one.

**Table 2:** Comparison of baseline characteristics of non-POD and POD groups

Variable	No POD (*n* = 247)	POD (*n* = 42)	*P*-value
Age	72.12 ± 6.77	73.50 ± 6.91	0.23
Male	136 (55.1%)	28 (66.7%)	0.18
Education levels			
6 years or less	198 (80.2%)	34 (81.0%)	1.00
More than 6 years	49 (19.8%)	8 (19.0%)	
BMI	24.29 ± 4.11	23.81 ± 4.52	0.47
ASA class			
Class II	65 (26.3%)	3 (7.1%)	0.01*
Class III	165 (66.8%)	38 (90.5%)	0.002*
Class IV	17 (6.9)	1 (2.4%)	0.49
Charlson comorbidity index	5.83 ± 1.94	6.55 ± 2.27	0.03*
Charlson comorbidity index > 5	120 (48.6%)	27 (64.3%)	0.07
Cardiovascular disease	215 (87.0%)	39 (92.9%)	0.44
Endocrine disease	180 (72.9%)	36 (85.7%)	0.09
Respiratory disease	25 (10.1%)	8 (19.0%)	0.11
Nervous system disease	56 (22.7%)	15 (35.7%)	0.08
CKD: 3a, 3b, 4, 5	91 (36.8%)	24 (57.1%)	0.01*
Modified IQCODE ≥ 3.42	29 (12.2%)	11 (28.2%)	0.01*
MoCA	19.47 ± 4.85	16.76 ± 6.76	0.01*
Previous Delirium	0 (0.0%)	2 (4.8%)	0.02*
PHQ-9 ≥ 7	13 (5.3%)	3 (7.1%)	0.71
Barthel ADL Index; score 0–70, moderately disabled	10 (4.1%)	1 (2.4%)	1.00
Barthel ADL Index; score 100	94.85 ± 9.95	94.04 ± 13.80	0.65
Barthel ADL Index; score < 100	93 (38.3%)	17 (40.5%)	0.86
IADL score	6.67 ± 1.79	5.90 ± 2.38	0.05
IADL score < 8 for women or score < 5 for men	63 (25.5%)	16 (38.1%)	0.09
Frailty	31 (12.6%)	7 (16.7%)	0.46
Quality of life at admission	0.85 ± 0.17	0.87 ± 0.17	0.72

* Indicates statistical significance (<0.05), Data are presented by n (%) or mean ± SD

*Abbreviations*: *ADL* activities of daily living, *ASA* American Association of Anesthesiologists, *BMI* body mass index, *CKD* chronic kidney diseases, *FRAIL* Fatigue, Resistance, Ambulation, Illnesses, and Loss of weight, *IADL* Lawton–Brody instrumental activities of daily living, *PHQ-9* 9-item Patient Health Questionnaire, *MoCA* Montreal cognitive assessment, *POD* postoperative delirium

Table 3 illustrates the outcomes of the participants whose cognitive status could be documented using the MoCA. POCD with POD groups (16 cases) were excluded from the analysis. At 3 months, a functional decline was found in 22.2% of the participants with 1-week POCD and 24.0% of those without POCD (*P* = 0.83). Compared with individuals without cognitive changes, the participants with POCD were more likely to suffer from unfavorable outcomes, namely, longer ICU and hospital stays and higher hospital costs. More specifically, the POCD group had a longer median stay in the ICU (2 days [0, 14] vs. 1 day [0, 11]; *P* = 0.01); a longer median hospital stay (11 days [1, 46] vs. 8 days [2, 42]; *P* = 0.01); and a higher median hospital expenditure (8973.43 USD [3481.69, 11 763.74] vs. 5913.62 USD [332.43, 19 567.33]; *P* < 0.001). Even though POCD with POD group was included in the analysis, the overall interpretations were the same (Appendix 2: Table S2).

**Table 3:** Postoperative outcomes of patients with POCD (includes only those participants who could be administered the MoCA 1 week after surgery to establish their POCD status)

Variable	No POCD (*n* = 148)	POCD (*n* = 43)	*P* value
Barthel ADL Index at 3 months	94.60 ± 11.62	92.22 ± 13.27	0.29
Declined basic ADL n (%); Barthel reduced ≥ 5 points	30 (23.8%)	9 (25.0%)	0.88
Declined IADL, n (%); score reduced ≥ 2 points	30 (24.0%)	8 (22.2%)	0.83
IADL score at 3 months	6.43 ± 1.9	6.25 ± 1.9	0.60
Worsened quality of life at 3 months, n (%)	37 (28.9%)	9 (25.0%)	0.68
Health utility, score	0.89 ± 0.14	0.89 ± 0.20	0.80
Frailty at 3 months, (3–5), n (%)	11 (8.7%)	6 (16.7%)	0.22
Readmission at 3 months, n (%)	16 (10.8%)	6 (14.0%)	0.59
Mortality at 3 months, n (%)	3 (2.0%)	1 (2.3%)	1.00
Hospital LOS, days; median (min, max)	8 (2, 42)	11 (1,46)	0.01*
Total cost, USD; median (min, max)	5913.62 (332.43, 19 567.33)	8973.43 (3481.69, 11 763.74)	< 0.001*
ICU LOS, days; median (min, max)	1 (0, 11)	2 (0, 14)	0.01*
Ventilator, days; median (min, max)	1 (0, 8)	1 (0, 3)	0.03*

^*^ Indicates statistical significance (< 0.05)

POCD includes participants who had MoCA administered 1 week after surgery to establish their POCD status but did not include patients with POD

*Abbreviations ADL* activities of daily living, *FRAIL* Fatigue, Resistance, Ambulation, Illnesses, and Loss of weight, *IADL* Lawton–Brody instrumental activities of daily living, *ICU* intensive care unit, *LOS* length of stay, *POCD* postoperative cognitive dysfunction, *ventilator* length of time on a ventilator

Another set of analyses was performed for patients with POD (Table 4). They were compared with people who did not develop POD, and the POCD with POD group was excluded from the analysis. At the 3-month follow-up, IADL decline was discovered in 22.7% of the participants without POD compared with 47.4% of the participants with POD (*P* = 0.03). The participants with POD were more likely to suffer from unfavorable outcomes, namely, a longer stay in the ICU, an increased hospital LOS, higher hospital expenditure, and a higher mortality rate. More specifically, the group with POD had a longer stay in the ICU (5 [0, 53] vs. 1 [0, 57]; *P* < 0.001); an increased hospital LOS (17 days [5, 70] vs. 8 days [1, 57]; *P* < 0.001); a higher hospital expenditure (11 501.52 USD [2,857.64, 86,953.34] vs. 5981.68 USD [332.04, 53 455.42]; *P* < 0.001); and a higher 3-month mortality rate (26.9% vs. 2.4%; *P* < 0.001). Table S3 shows the results without excluding the POCD with POD group. The overall interpretations were similar, except for no difference in IADL decline at month 3 between the POD group and the non-POD one.

**Table 4:** Postoperative outcomes of patients with POD

Variable		No POD (*n* = 247)	POD (*n* = 26)	*P* value
Barthel ADL Index at 3 months	93.69 ± 12.68		87.63 ± 19.95	0.06
Declined basic ADL n (%); score reduced ≥ 5 points	49 (25.1%)		6 (31.6%)	0.58
Declined IADL score 2, n (%); score reduced ≥ 2 points	44 (22.7%)		9 (47.4%)	0.03*
IADL score at 3 months	6.33 ± 1.89		5.10 ± 2.42	0.04*
Worsened quality of life at 3 months, n (%)	57 (28.9%)		7 (36.8%)	0.60
Health utility, score	0.90 ± 0.15		0.86 ± 0.19	0.33
Frailty at 3 months, (3–5), n (%)	22 (11.3%)		3 (15.8%)	0.47
Readmission at 3 months, n (%)	32 (13.2%)		6 (25.0%)	0.13
Mortality at 3 months, n (%)	6 (2.4%)		7 (26.9%)	< 0.001*
Hospital LOS, days; median (min, max)	8 (1, 57)		17 (5, 70)	< 0.001*
Total cost, USD; median (min, max)	5981.68 (332.04, 53 455.42)		11 501.52 (2857.64, 86 953.34)	< 0.001*
ICU LOS, days; median (min, max)	1 (0, 57)		5 (0, 53)	< 0.001*
Ventilator, days; median (min, max);	1 (0, 11)		1 (0, 57)	< 0.001*

^*^ Indicates statistical significance (< 0.05)

POD includes participants who had DSM-5 administered to establish their POD status but did not include patients with POCD

*Abbreviations***:***ADL* activities of daily living, *FRAIL* Fatigue, Resistance, Ambulation, Illnesses, and Loss of weight, *IADL* Lawton–Brody instrumental activities of daily living, *ICU* intensive care unit, *LOS* length of stay, *POD* postoperative delirium; ventilator, length of time on a ventilator

Table 5 presents the factors associated with functional decline at the 3-month follow-up. The multivariate model included relevant covariates, namely age, gender, surgery type, POCD, and postoperative complications (POD, pneumonia, acute kidney injury (AKI), arrhythmia, and reoperation). At that visit, patients with POD had a significantly higher risk of functional decline (adjusted RR 2.33; 95% CI, 1.15–4.71; *P* = 0.02). Even though patients with POCD had a greater risk of developing a functional decline, there was no statistical significance (adjusted RR 1.01 [0.49–2.09]; *P* = 0.96). No factors other than POD were related to reduced function after surgery.

**Table 5:** Factors associated with functional decline 3 months after surgery

Variable	Univariate analysis Crude RR (95% CI)	*P-*value	Multivariate analysis Adjusted RR^a^ (95% CI)	*P*-value
Cognition				
No cognitive change (*n* = 163)	1		1	
POCD (*n* = 37)	0.96 (0.51–1.81)	0.91	1.01 (0.49–2.09)	0.96
POD (*n* = 26)	2.44 (1.63–3.66)	< 0.05*	2.33 (1.15–4.71)	0.02*
POCD with POD (*n* = 14)	1.49 (1.01–2.21)	0.14	1.33 (0.50–3.55)	0.56
Preoperative frailty				
(*n* = 31)	0.99 (0.55–1.79)	1.00	–	–
Preoperative Barthel ADL Index; score ≤ 70 (*n* = 9)	1.14 (0.44–2.95)	0.72	–	–
Preoperative Modified IQCODE				
≥ 3.42 (*n* = 37)	0.94 (0.53–1.67)	0.85	–	–
Female				
(*n* = 100)	1.05(0.70–1.56)	0.89	1.22 (0.73–2.04)	0.45
Age ≥ 75 years (*n* = 85)	1.14 (0.76–1.71)	0.55	1.04 (0.62–1.76)	0.88
Charlson comorbidity index				
≥ 5 (*n* = 183)	1.24 (0.75–2.06)	0.41	–	–
Baseline PHQ-9				
≥ 7 (*n* = 12)	1.15 (0.50–2.62)	0.75	–	–
Cardiac surgery (*n* = 136)	1.14 (0.76–1.71)	0.57	0.73 (0.44–1.29)	0.26
Postoperative complications (*n* = 240)				
Pneumonia				
(*n* = 12)	1.78 (0.97–3.25)	0.19	0.53 (0.14–1.94)	0.34
Acute kidney injury				
(*n* = 24)	2.25 (1.49–3.37)	< 0.05*	1.93 (0.77–4.84)	0.16
Arrhythmia				
(*n* = 48)	1.60 (1.06–2.41)	0.01*	0.93 (0.49–1.76)	0.82
Urinary tract infection				
(*n* = 6)	1.74 (0.76–3.98)	0.36	–	–
Convulsion				
(*n* = 3)	1.14 (0.22–5.74)	1.00	–	–
Reoperation				
(*n* = 14)	2.08 (1.26–3.43)	0.03*	1.19 (0.35–4.04)	0.78

^*^ Indicates statistical significance (< 0.05)

POCD includes participants who had MoCA administered 1 week after surgery to establish their POCD status but did not include patients with POD

POD includes participants who had DSM-5 administered to establish their POD status but did not include patients with POCD

POCD with POD includes participants who had MoCA administered 1 week after surgery and DSM-5 administered to establish their POCD status and POD status, respectively

^a^The model adjusted for gender, age, type of surgery, pneumonia, acute kidney injury, arrhythmia, and reoperation

*Abbreviations***:***Barthel ADL* Barthel activities of daily living, *POCD* postoperative cognitive dysfunction, *POD* postoperative delirium

## Discussion

To date, debates about whether POCD and POD are the same or separate entities have had inconclusive results [42–44]. Several POD studies have shown convincing evidence of subsequent functional decline after surgery [45–48]. However, there have been inconclusive results on the association between POCD and postoperative functional decline. In the present study, patients experiencing POCD at 1 week were not associated with a decrease in IADLs 3 months after surgery, whereas the POD group showed an increased risk of functional decline. Previous evidence from cardiac and noncardiac surgery showed that the appearance of POCD between the first and third months after surgery could cause a decrease in IADLs [20, 25, 41]. Furthermore, patients with POCD 1 week after noncardiac surgery had an increased risk of leaving the labor market due to a decrease in function compared with non-POCD patients [22]. In contrast, another study did not report any differences in the 3-month functional outcomes for POCD patients after undergoing cardiac surgery [19]. That study proposed that the preexisting cognitive status of a patient was the main factor that affected postoperative functional changes. Interestingly, participants in the present study who developed POCD were more likely to have a better cognitive status than the non-POCD patients. This finding might contribute to the lesser adverse effects of POCD on functional outcomes. However, another possibility could be since the definition of POCD utilized in this study using the drop in brief assessment might not be sensitive enough to classify patients with this condition.

Regarding other negative consequences of postoperative cognitive disorders, POCD in this study did not increase the risk of death during the first three postoperative months. In contrast, POD did have a significant risk of increased mortality. A previous study reported that POCD patients at discharge were more likely to die before their 3-month follow-up [4]. The population of that work was similar to that of this study but had an even younger age. Other investigations found that POCD was associated with both short- and long-term mortality [21, 22, 44]. The sample size of the present study may have been too small to demonstrate a difference in short-term mortality. Therefore, the association between POCD and mortality might need a larger sample size with a longer-term follow-up.

Furthermore, we explored the relationship between POCD and healthcare utilization. Interestingly, POCD patients were more likely to use the healthcare system than those who did not have the condition. The increased utilization was evidenced by the more prolonged ICU and hospital stays and the higher costs. Furthermore, a higher proportion of patients with POCD were readmitted to the hospital than those without POCD, although the difference was nonsignificant. The result of this study is consistent with a previous investigation of increased healthcare costs during the 1-year postoperative period [21]. Our findings highlight the consequences of POCD: although clinically subtle, POCD has noticeable effects on healthcare system expenditure. Considering the increasing magnitude of this disease as the population gradually ages, an appropriate strategy would be to have multidisciplinary teams handle the condition.

Unlike other studies [38, 41], the present work did not find any associations between a decrease in IADLs and several preoperative and postoperative factors (age of 75 years or older, multiple comorbidities, preoperative frailty, preexisting cognitive impairment, depression, dependence at baseline, and postoperative complications). This result could be because the current research focused on elective surgery and had fairly robust participants in terms of their functional status before surgery. Although a substantial proportion of patients exhibited some degree of cognitive impairment, the condition was not associated with decreased function at the 3-month follow-up. This observation suggests that mild cognitive impairment might not lead to a significant decline in function after an operation. In this study, preoperative frailty status did not contribute to the decline in IADLs. This finding differed from those of several other studies, which reported a significant relationship between baseline frailty and poor functional outcomes [49, 50]. The presence of preoperative frailty might predict unfavorable results among older-old and oldest-old patients [49]. As most of the participants in our work were in the young-old age group, their frailty status might not have made a significant contribution to postoperative functional decline.

The study highlighted the consequences of POCD and POD on clinical outcomes and healthcare utilization. The research has several strengths and limitations. One strength was that participants were prospectively screened for POCD and POD, with physicians confirming positive findings through clinical evaluations. This approach minimized misclassifications of the conditions. Even though POD was assessed for a shorter period after noncardiac surgery and could potentially lead to under-recognition of POD, geriatricians comprehensively reviewed through medical records would minimize the issue. As to the neuropsychological testing, we utilized a brief cognitive test, MoCA, which covers multiple cognitive domains and is easy to use in daily practice. Still, this could be both a limitation and a strength. The limitation might be that MoCA is less comprehensive than multiple neuropsychological batteries. However, a brief test like MoCA can identify patients with adverse postoperative outcomes. It is also more practical than multiple batteries for wide adoption in clinical practice, particularly in resource-limited settings where neuropsychologists are scarce.

MoCA has recently been applied in several studies of POCD [13, 51]. Unfortunately, a considerable proportion of patients in those studies did not have MoCA performed postoperatively, leading to selection bias. By contrast, our analyses also included patients without MOCA reassessments and identified no significant differences from those with MoCA reassessments even though 28% of total participants were missing from the MoCA tests for the primary outcome assessments for several reasons, including death, medically unstable conditions, and refusing participation. Additionally, of 289, 21% had no functional outcome at month three due to death and loss to follow-up. Another consideration is that the sample size in our study could have lacked sufficient power to detect some outcomes of the POCD group (for example, functional outcome and mortality). Lastly, healthcare costs were underestimated by the current investigation because direct nonmedical expenditures were not assessed.

## Conclusions

We showed that patients with POCD and those with POD were more likely to utilize healthcare services. Furthermore, POD increases the risk of developing functional decline after surgery. Therefore, the use of preventive measures, early detection, and appropriate interventions for POCD and POD among older surgical patients might be a critical factor in improving the standard of care of patients and reducing the burden on families and society.

## Supplementary Information

**Additional file 1.**

**Additional file 2**.

**Additional file 3.**

**Additional file 4.**

## Data Availability

The datasets used and/or analyzed during the current study are available from the corresponding author on reasonable request.

## Abbreviations

ASA: American Association of Anesthesiologists
Barthel ADL: Barthel activities of daily living
BMI: Body mass index
CKD: Chronic kidney diseases
FRAIL: Fatigue, resistance, ambulation, illness, and loss of weight
IADL: Lawton–Brody instrumental activities of daily living
MoCA: Montreal cognitive assessment
POCD: Postoperative cognitive dysfunction
POD: Postoperative delirium
