# Supplementary material for: Postoperative cognitive dysfunction in older surgical patients associated with increased healthcare utilization: a prospective study from an upper-middle-income country
Source: BMC Geriatr. 2022 Mar 16;22:213. doi: 10.1186/s12877-022-02873-3 (PMC8925052; doi:10.1186/s12877-022-02873-3)
Supplement: Supplementary file 1 — Additional file 1. [file 12877_2022_2873_MOESM1_ESM.docx]

**Appendix 2.**

**Table S1: Demographic data**

| Variable | Total  (n = 289) | No cognitive change  (n = 204) | POCD without POD  (n = 43) | POD  (n = 42) |
| --- | --- | --- | --- | --- |
| Age | 72.3 ± 6.7 | 72.4 ± 6.6 | 70.7 ± 7.3 | 73.5 ± 6.9 |
| Male | 164 (56.7%) | 113 (55.4%) | 23 (53.5%) | 28 (66.7%) |
| Education levels |  |  |  |  |
| 6 years or less | 232 (80.3%) | 160 (78.4%) | 38 (88.4%) | 34 (81.0%) |
| More than 6 years | 57 (19.7%) | 44 (21.6%) | 5 (11.6%) | 8 (19.0%) |
| BMI | 24.33 ± 4.15 | 24.32 ± 4.05 | 24.13 ± 4.44 | 23.81 ± 4.52 |
| ASA class |  |  |  |  |
| Class II | 68 (23.5%) | 60 (29.4%) | 5 (11.6%) | 3 (7.1%) |
| Class III | 203 (70.2%) | 132 (64.7%) | 33 (76.7%) | 38 (90.5%) |
| Class IV | 18 (6.2%) | 12 (5.9) | 5 (11.6%) | 1 (2.4%) |
| Charlson comorbidity index | 5.91 ± 1.98 | 5.92 ± 1.98 | 5.40 ± 1.70 | 6.55 ± 2.27 |
| Charlson comorbidity index > 5 | 147 (50.9%) | 102 (50.0%) | 18 (41.9%) | 27 (64.3%) |
| Cardiovascular disease | 254 (87.9%) | 174 (85.3%) | 41 (95.3%) | 39 (92.9%) |
| Endocrine disease | 216 (74.7%) | 147 (72.1%) | 33 (76.7%) | 36 (85.7%) |
| Respiratory disease | 33 (11.4%) | 22 (10.8%) | 3 (7.0%) | 8 (19.0%) |
| Nervous system disease | 71 (24.6%) | 49 (24.0%) | 7 (16.3%) | 15 (35.7%) |
| CKD: 3a, 3b, 4, 5 | 115 (39.8%) | 73 (35.8%) | 18 (41.9%) | 24 (57.1%) |
| Modified IQCODE ≥ 3.42 | 40 (14.5%) | 26 (13.3%) | 3 (7.1%) | 11 (28.2%) |
| MoCA | 19.1 ± 5.2 | 19.0 ± 4.8 | 21.5 ± 4.8 | 16.7 ± 6.3 |
| Variable | **Total**  **(n = 289)** | **No cognitive change**  **(n = 204)** | **POCD without POD**  **(n = 43)** | **POD**  **(n = 42)** |
| PHQ-9 ≥ 7 | 16 (5.5%) | 8 (3.9%) | 5 (11.6%) | 3 (7.1%) |
| Barthel ADL Index; score  0–70, moderately disabled | 11 (3.9%) | 9 (4.5%) | 1 (2.3%) | 1 (2.4%) |
| Barthel ADL Index score; score 100 | 94.7 ± 10.2 | 94.9 ± 9.6 | 94.9 ± 8.2 | 94.2 ± 14.5 |
| Barthel ADL Index;  score < 100 | 110 (38.6%) | 74 (37.0%) | 19 (44.2%) | 17 (40.5%) |
| IADL score | 6.56 ± 1.90 | 6.66 ± 1.76 | 6.69 ± 1.94 | 6.00 ± 2.38 |
| IADL score < 8 for women or score < 5 for men  Frailty | 79 (27.7%)  38 (13.1%) | 53 (26.5%)  22 (10.8%) | 10 (23.3%)  9 (20.9%) | 16 (38.1%)  7 (16.7%) |
| Quality of life at admission | 0.86 ± 0.17 | 0.86 ± 0.16 | 0.87 ± 0.16 | 0.86 ± 0.18 |

Data are presented by n (%) or mean ± SD.

**Abbreviations:** ADL, activities of daily living; ASA, American Association of Anesthesiologists; BMI, body mass index; CKD, chronic kidney diseases; FRAIL, Fatigue, Resistance, Ambulation, Illnesses, and Loss of weight; IADL, Lawton–Brody instrumental activities of daily living; PHQ-9, 9-item Patient Health Questionnaire; MoCA, Montreal cognitive assessment; POCD, postoperative cognitive dysfunction.
