# Supplementary material for: Postoperative cognitive dysfunction in older surgical patients associated with increased healthcare utilization: a prospective study from an upper-middle-income country
Source: BMC Geriatr. 2022 Mar 16;22:213. doi: 10.1186/s12877-022-02873-3 (PMC8925052; doi:10.1186/s12877-022-02873-3)
Supplement: Supplementary file 2 — Additional file 2. [file 12877_2022_2873_MOESM2_ESM.docx]

**Table S2: Postoperative outcomes of patients with POCD** (including only those participants who had MoCA administered 1 week after surgery to establish their POCD status)

| Variable | No POCD (n = 148) | POCD at 1 week (n = 59) | *P*-value |
| --- | --- | --- | --- |
| Barthel ADL Index at 3 months | 94.6 ± 11.6 | 92.7 ± 13.6 | 0.34 |
| Declined basic ADL, n (%); score reduced ≥ 5 points | 30 (23.8%) | 13 (26.5%) | 0.16 |
| Declined IADL, n (%); score reduced ≥ 2 points | 30 (24.0%) | 11 (22.4%) | 0.83 |
| IADL score at 3 months | 6.43 ± 1.9 | 6.1 ± 2.0 | 0.33 |
| Worsened quality of life at 3 months, n (%) | 37 (28.9%) | 13 (26.5%) | 0.75 |
| Quality of life, score | 0.89 ± 0.14 | 0.89 ± 0.18 | 0.96 |
| Frailty at 3 months, n (%) | 11 (8.7%) | 9 (18.4%) | 0.07 |
| Readmission at 3 months, n (%) | 16 (10.8%) | 10 (16.9%) | 0.23 |
| Mortality at 3 months, n (%) | 3 (2.0%) | 2 (3.4%) | 0.63 |
| Hospital LOS, days; median  (min, max) | 8 (2, 42) | 11 (1, 61) | < 0.001* |
| Total cost, USD; median  (min, max) | 5913.62  (332.43, 19 567.33) | 9045.37  (3408.69, 31 647.45) | < 0.001* |
| ICU LOS, days; median  (min, max) | 1 (0, 11) | 2 (0, 14) | < 0.001* |
|  |  |  |  |
| Variable | **No POCD (n = 148)** | **POCD at 1 week (n = 59)** | ***P*-value** |
| Ventilator, days; median  (min, max) | 1 (0, 8) | 1 (0, 6) | 0.01* |

* Indicates statistical significance (< 0.05)

**Abbreviations:** ADL, activities of daily living; FRAIL, Fatigue, Resistance, Ambulation, Illnesses, and Loss of weight; IADL, Lawton–Brody instrumental activities of daily living; ICU, intensive care unit; LOS, length of stay; POCD, postoperative cognitive dysfunction; ventilator, length of time on a ventilator.
