# Supplementary material for: Postoperative cognitive dysfunction in older surgical patients associated with increased healthcare utilization: a prospective study from an upper-middle-income country
Source: BMC Geriatr. 2022 Mar 16;22:213. doi: 10.1186/s12877-022-02873-3 (PMC8925052; doi:10.1186/s12877-022-02873-3)
Supplement: Supplementary file 3 — Additional file 3. [file 12877_2022_2873_MOESM3_ESM.docx]

**Table S3: Postoperative outcomes of patients with POD**

| Variable | No POD (n = 247) | POD (n = 42) | *P-*value |
| --- | --- | --- | --- |
| Barthel ADL Index at 3 months | 93.69 ± 12.68 | 90.15 ± 17.80 | 0.29 |
| Declined basic ADL n (%); score reduced ≥ 5 points | 49 (25.1%) | 10 (31.3%) | 0.52 |
| Declined IADL score 2, n (%); score reduced ≥ 2 points | 44 (22.7%) | 12 (37.5%) | 0.08 |
| IADL score at 3 months | 6.33 ± 1.89 | 5.37 ± 2.36 | 0.04* |
| Worsened quality of life at 3 months, n (%) | 57 (28.9%) | 11 (34.4%) | 0.68 |
| Quality of life, score | 0.90 ± 0.15 | 0.88 ± 0.16 | 0.58 |
| Frailty at 3 months, n (%) | 22 (11.3%) | 6 (18.8%) | 0.25 |
| Readmission at 3 months, n (%) | 32 (13.2%) | 10 (25.0%) | 0.06 |
| Mortality at 3 months, n (%) | 6 (2.4%) | 8 (19.0%) | < 0.001* |
| Hospital LOS, days; median  (min, max) | 8 (1, 57) | 14 (5, 70) | < 0.001* |
| Total cost, USD; median  (min, max) | 5981.68  (332.04, 53 455.42) | 10 326.79  (2793.27, 84 994.69) | < 0.001* |
| ICU LOS, days; median  (min, max) | 1 (0, 57) | 4 (0, 53) | < 0.001* |
| Ventilator, days; median  (min, max) | 1 (0, 11) | 1 (0, 53) | < 0.001* |

* Indicates statistical significance (< 0.05)

**Abbreviations:** ADL, activities of daily living; FRAIL, Fatigue, Resistance, Ambulation, Illnesses, and Loss of weight; IADL, Lawton–Brody instrumental activities of daily living; ICU, intensive care unit; LOS, length of stay; POD, postoperative delirium; ventilator, length of time on a ventilator.
