# Supplementary material for: Postoperative cognitive dysfunction in older surgical patients associated with increased healthcare utilization: a prospective study from an upper-middle-income country
Source: BMC Geriatr. 2022 Mar 16;22:213. doi: 10.1186/s12877-022-02873-3 (PMC8925052; doi:10.1186/s12877-022-02873-3)
Supplement: Supplementary file 4 — Additional file 4. [file 12877_2022_2873_MOESM4_ESM.docx]

**Appendix 1**

**Figure S1: Timeline for POCD and POD diagnosis**

Preoperative MoCA

Surgery

CAM-ICU D1-D5

Positive Negative

DSM-5

Positive Negative

POD No POD No POD

Postoperative D5-D9

MoCA No MoCA

Decreased score ≥2 Decreased score <2 Chart review

DSM-5 DSM-5

Positive Negative Positive Negative

POD (with POCD) POCD No POCD, no POD POD No POD

**Abbreviations:** MoCA, Montreal cognitive assessment; CAM-ICU, confusion assessment method for the intensive care unit; DSM-5, Diagnostic and Statistical Manual of Mental Disorders; POCD, postoperative cognitive dysfunction; POD, postoperative delirium
